# High-intensity Focused Ultrasound-A New Choice to Conduct Pulmonary Artery Denervation

**DOI:** 10.1007/s12265-024-10531-9

**Published:** 2024-07-06

**Authors:** Yonghui Xie, Taoyue Yao, Xiaogang Zhu, Fan Yang, Haoqin Fan, Shirui Cao, Huaiyang Chen, Manzhen Liao, Yuanxi Xia, Jinqiao Liu, Zhenghui Xiao, Zhou Yang, Yunbin Xiao

**Affiliations:** 1 Academy of Pediatrics, University of South China, Changsha, 410007 China; 2 Department of Cardiology, The Affiliated Children’s Hospital of Xiangya School of Medicine, Central South University (Hunan children’s hospital), Changsha, 410007 China; 3 Department of Ultrasound, The Affiliated Children’s Hospital of Xiangya School of Medicine, Central South University (Hunan children’s hospital), Changsha, 410007 China; 4 Department of Obstetrics and Gynecology, Third Xiangya Hospital, Central South University, Changsha, 410013 China; 5 Department of Cardiovascular Medicine, Second Xiangya Hospital, Central South University, Changsha, 410011 China; 6 Class 2115, Changsha Yali High School, Changsha, 410007 China; 7 Department of Anesthesiology, The Affiliated Children’s Hospital of Xiangya School of Medicine, Central South University (Hunan children’s hospital), Changsha, 410007 China; 8 Department of Pediatric Intensive Care, The Affiliated Children’s Hospital of Xiangya School of Medicine, Central South University (Hunan children’s hospital), Changsha, 410007 China

**Keywords:** Pulmonary Arterial Hypertension, Pulmonary Artery Denervation, High-intensity Focused Ultrasound, Sympathetic Nerves

## Abstract

This research aimed to explore whether high-intensity focused ultrasound (HIFU) could conduct pulmonary artery denervation (PADN). HIFU was performed in pulmonary arteries of 6 normotensive rabbits at dose of 250W, 6 times for each rabbit, and an additional 6 rabbits served as controls. Then ATEPH was induced in both groups by intravenous infusion of autogeneic thrombus. Hemodynamics and ultrasonography parameters were measured by right heart catheter and echocardiography pre- and post-establishment of ATEPH models in both groups. Histological analysis and immunohistochemistry of tyrosine hydroxylase (TH) were also performed. After PADN procedures, 5 rabbits were successfully conducted PADN, of which ablation zone was also observed in right auricle or right lung in 4 rabbits. Ablation zone was detected only in right lung in 1 rabbit. Compared with control group, milder right heart hemodynamic changes were found in PADN group, accompanied by improved ultrasound parameters in PADN group. HIFU can acutly damage SNs around pulmonary artery successfully, which may be a new choice to conduct PADN. However, the accuracy of HIFU with PADN needs to be improved.

**Graphical Abstract:**

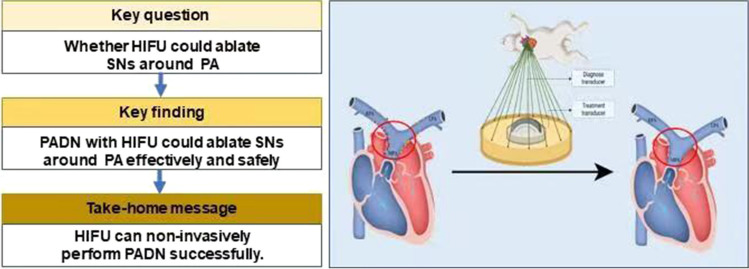

## Introduction

Pulmonary arterial hypertension (PAH) is a complex and fatal disease with increasing pulmonary vascular resistance and right heart failure. Vasoconstriction plays a vital role in the progression of PAH, which makes amelioration of vasoconstriction become the most important target of combined targeted medicine [1]. However, not all patients can benefit from target medicine or tolerate side effects of target medicine, which inspires to find more suitable therapy [2].

Along pulmonary artery (PA) exists a great deal of sympathetic nerves (SNs), especially around main pulmonary artery (MPA) and PA bifurcation [3]. Previous studies demonstrated that over-activation of SNs promotes development of PAH in animal models and patients [4]. When SNs damaged by pulmonary artery denervation (PADN), PAH patients and animal models can be relieved significantly [4, 5]. Recent decades, preclinical and clinical studies attempted to explore the best way to perform PADN, such as chemical [6], surgical [7] and catheter-based [8] ways. These trials demonstrated that after SNs being destroyed, hemodynamic status and quality of life can be improved obviously. Catheter-based denervation has conducted clinical studies successfully, while some patients cannot perform PADN successfully because of unbearable chest pain during PADN procedure [9]. Besides, there are some possible path-related complications, such as arterial perforation, vasospasm, femoral artery pseudoaneurysms, and subcutaneous hematoma [10]. Moreover, there is high risk of general anesthesia in PAH patients with poor condition, which promotes researchers to search for new ways to perform PADN.

High-intensity focused ultrasound (HIFU) can non-invasively deliver acoustic energy to deep sites of body without causing obvious damages to adjacent tissues [11]. HIFU has been applied to ablate liver tumor [12], prostatic tumor [13], gynecological diseases [14] and so on, which showed non-invasion, safety and relative convenience. Recently, a investigation has proved application of HIFU could externally denervate renal sympathetic nerves effectively and safely in animal models via disrupting renal sympathetic nerves and diminishing sympathetic tone, which emerged as a promising device-based therapy for hypertension [10]. Studies also demonstrated that PADN improved hemodynamic status in patients and animal models with PAH [3, 4, 15–17]. However, whether HIFU could externally ablate SNs around pulmonary artery had not been explored.

We therefore explored PADN effect of HIFU in rabbit models, and we found HIFU can acutly damage SNs effectively and safely, which may be a new choice to conduct PADN.

## Methods

The experiment protocol was approved by the Institutional Animal Care and Use Committee of Hunan Children’s Hospital and in accordance with the Guide for the Care and Use of Laboratory Animals (National Research Council).

### Animal Preparation

**Fig. 1:**
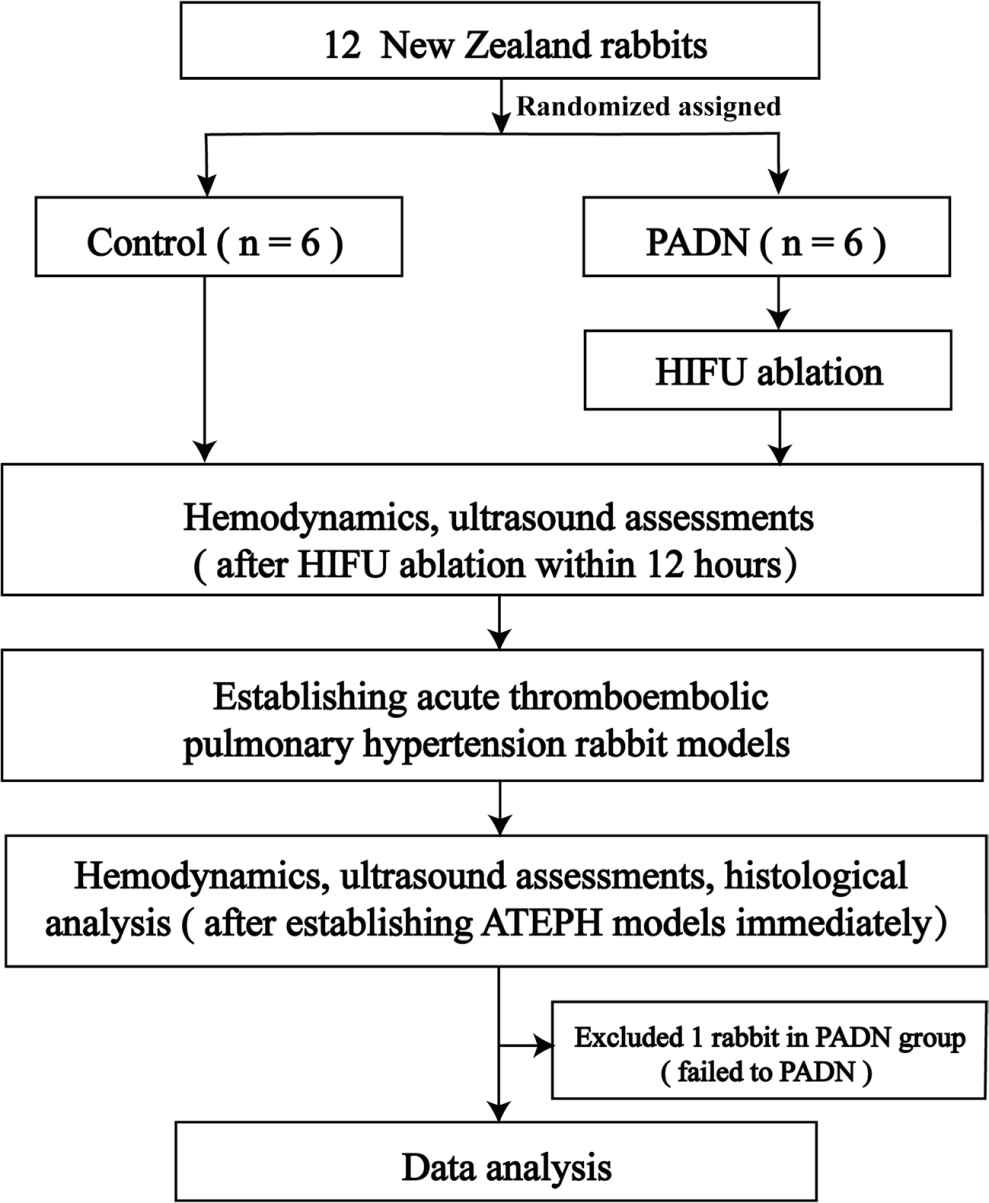
Study flowchart. 12 New Zealand rabbits were randomly divided into control group and PADN group. After PADN procedure, hemodynamics and ultrasonography parameters were measured by right heart catheter and echocardiography pre- and post-establishment of ATEPH models in both groups within 12 h. Then MPA and bifurcation of PA were harvested immediately for histological analysis and immunohistochemistry of tyrosine hydroxylase when right heart catheter finished. PADN = pulmonary artery denervation group. HIFU = high-intensity focused ultrasound

A total of 12 New Zealand rabbits, of either sex, weighing 2000 to 2500 g, were randomly divided into control group and PADN group. All rabbits were anesthetized by intravenous injection of pentobarbital sodium (30 mg/Kg). Anesthesia was maintained by intravenous injection of pentobarbital sodium (10 mg/Kg) every hour. Depilatory creams were used to remove the hairs on the chest of rabbits. After PADN procedure, hemodynamics and ultrasonography parameters were measured by right heart catheter and echocardiography pre- and post-establishment of ATEPH models in both groups within 12 h. Then MPA and bifurcation of PA were harvested immediately for histological analysis and immunohistochemistry of tyrosine hydroxylase (TH) when right heart catheter finished (Fig. 1).

### HIFU Ablation

**Fig. 2:**
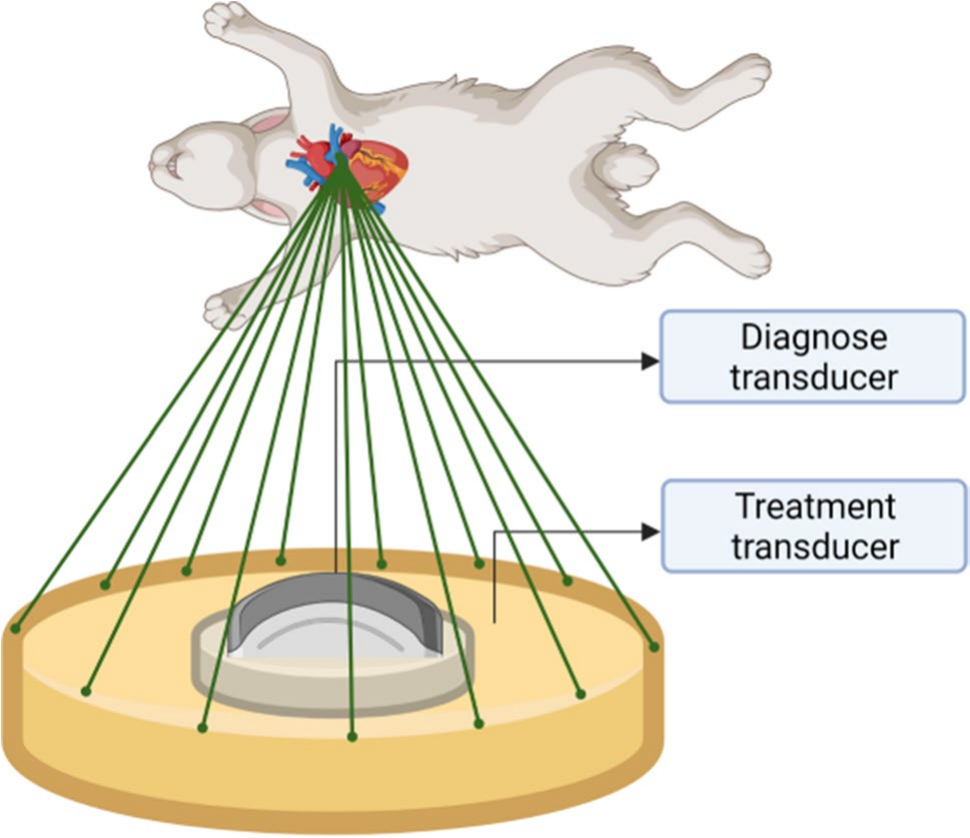
Illustration of PADN procedure with HIFU. With rabbit prone at a 45 degree angle, ablation sites were confirmed via diagnose transducer, and then acoustic wave was focused on PA by treatment transducer

HIFU procedure was conducted with JC-200 focused ultrasound tumor therapeutic system (Chongqing Haifu Tech Co., Ltd., Chongqing, China). A diagnostic transducer (My-Lab70, Esaote, Italy) was aligned coaxially to therapeutic transducer to locate target tissues (Fig. 2), and ultrasound energy was delivered via a spherically curved therapeutic transducer, with focal length of 140 mm. After rabbits prone, the space between chest skin surface and therapeutic transducer filled with degassed water. By adjusting the diagnostic transducer in three dimensions, the focus was moved to target, and ablation sites was also confirmed on ultrasound screen. Three ablation sites (right wall, left wall and middle wall of distal MPA) were located with parasternal short axis view (PSAX). Each target was ablated in dose of 250 W × 2 s, and then repeat the procedure after 2 s intermittence, eventually each rabbit was ablated six times with HIFU procedure.

### Right Heart Catheterization

Hemodynamic measurements were conducted by right heart catheterization in all rabbits. Under ultrasound guidance, 5-French flow-directed catheter was penetrated into right jugular vein, then put in right ventricle to measureright ventricle systolic pressure (RVSP), right ventricle diastolic pressure (RVDP) and mean right ventricle pressure (mRVP). Similarly, right femoral vein catheterization was performed, then 2 ml of venous blood were drawn from catheter. The blood clot was prepared by incubating venous blood for 60 min on sterile plates with 50 U thrombin and then blood clot was cut into 2 mm × 2 mm × 2 mm size and suspended in 5 ml saline solution. Under guideline of ultrasound, thrombus suspension was quickly injected via right femoral vein catheter to make autogeneic thrombus for establishing ATEPH rabbit models [18].

### Ultrasound Assessments

All rabbits had transthoracic two-dimensional (2-D), M-mode and Doppler imaging using a Philips EPIQ 7C Ultrasound System (Philips Corporation, American) with a X12-8 transducer, frequency ranging from 8 ~ 12 MHz. Pulmonary artery diameter (PAD) was measured from PSAX. A pulsed wave (PW) Doppler was used to measure pulmonary artery acceleration time (PAAT) in PSAX view. Four chamber dimensions of heart were measured from apical four-chamber view (A4C), then placing the M-mode cursor through the lateral annulus of the tricuspid valve, measuring tricuspid annular plane systolic excursion (TAPSE). All measurements were carried out according to the guidelines for the American Society of Echocardiography [19]. An experienced sonographer conducted a blind study of all rabbits.

### Histopathology

MPA and bifurcation of PA were harvested, and the PA tissues were fixed with 4% paraformaldehyde for 24 h, then embedded into paraffin, sliced into 5-µm-thick section and subjected to hematoxylin and eosin (HE) staining for histomorphometric analysis. PA sections were also stained successively with alcian blue, hematoxylin and so on according to instructions of Movat-Russell modified Pentachrome stains. Injuries of SNs adjacent to PA were evaluated with grade 0–4 by a standard scoring system of Sakakura et al. (Grade 0 = no obvious injury, Grade 1 = minimal injury, Grade 2 = mild injury, Grade 3 = moderate injury, Grade 3 = severe injury) [20, 21].

### Immunohistochemistry

PA slices from each group were prepared for further immunohistochemical stains against tyrosine hydroxylase (TH) protein, a marker for recognition of nerve fascicles. In brief, after PA slices being dewaxed, all slices were put in a microwave oven for antigen repaired, and polyclonal sheep anti rabbit TH (dilution 1:200, NB300-110SS, NOVUS) was added to these slices, incubated overnight at 4 °C in a wet box, then, secondary antibody were added to slices. Finally, slices were observed by microscopy. The distribution and intensity of immunostaining against TH was evaluated with grade 0–3 (0 = no reaction, 1 = patchy/very weak reaction, 2 = weak to moderate reaction, 3 = strong reaction) [20, 21].

### Statistical Analysis

All data are expressed as mean ± standard deviation (SD) and performed using Kolmogorov–Smirnov and Shapiro–Wilk tests. Data before and after establishing ATEPH rabbits in the same group were analyzed with paired t test. Data were analyzed by two-sample t test or Mann–Whitney U test to determine differences between control group and PADN group. Statistical significance was defined as a 2-sided *p* value < 0.05. All analyses were performed using SPSS 26.0.

## Results

### Animal Outcomes

After PADN procedures, 5 rabbits were successfully conducted PADN, of which the ablation zone was also observed in right auricle or right lung in 4 rabbits. And ablation zone was detected only in right lung in 1 rabbit.

### Right Heart Catheterization

**Fig. 3:**
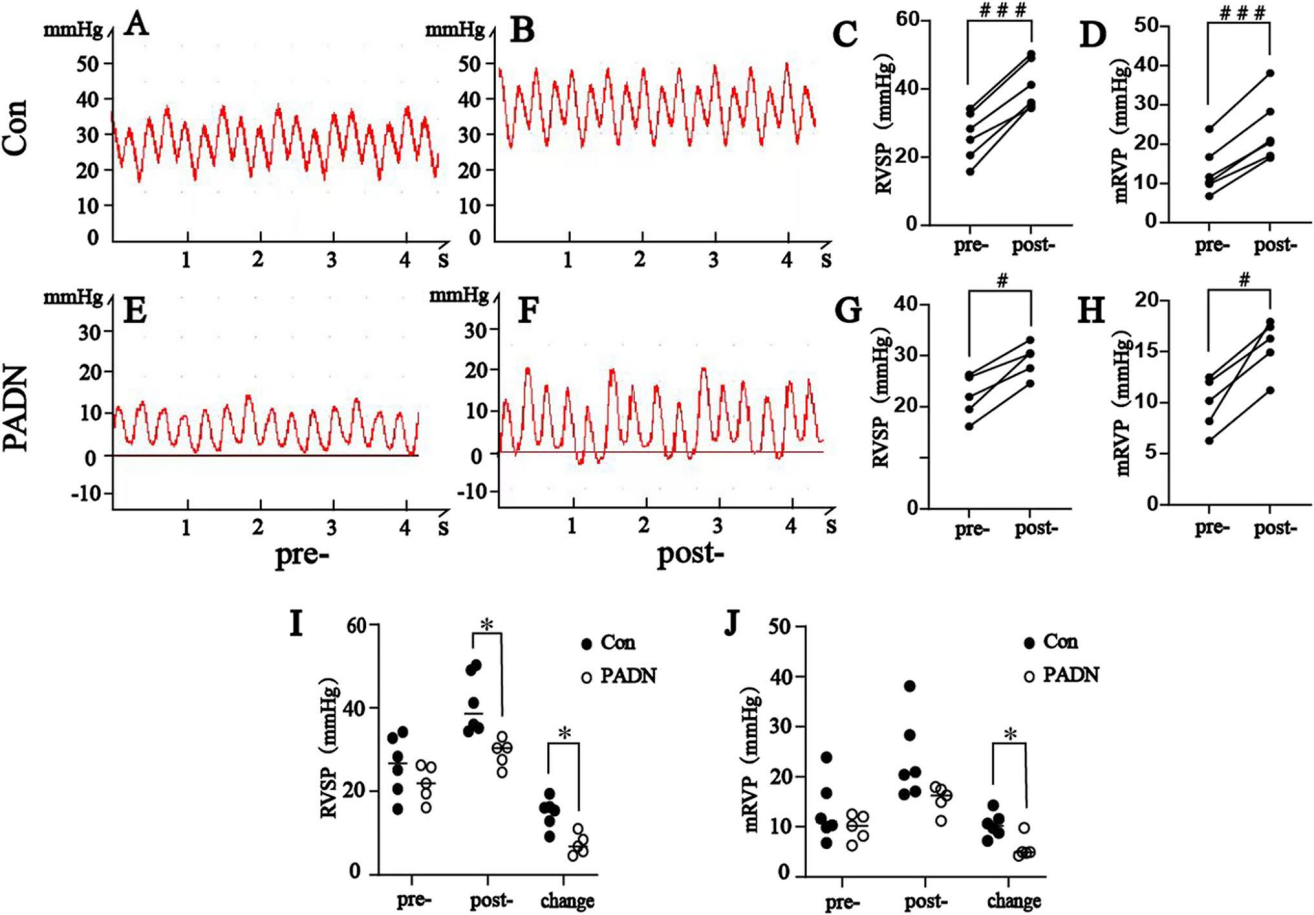
Hemodynamic parameters in control group (**A-D**) and PADN group (**E–H**) before and after establishing ATEPH models. Comparisons of RVSP (**I**) and mRVP (**J**) between control group and PADN group before and after establishing ATEPH models. Con = control group; PADN = pulmonary artery denervation group; RVSP = right ventricle systolic pressure; mRVP = Mean right ventricle pressure. Comparison in the same group before and after establishing ATEPH rabbits: ^#^ *P* < 0.05, ^###^*P* < 0.001; Comparison between control group and PADN group: ^*^ *P* < 0.05

Acute thromboembolic pulmonary hypertension models were established in both groups. Compared with the pre-ATEPH RVSP values, all rabbits showed significantly increased RVSP (*P* < 0.05, Table 1), post-ATEPH RVSP increased over 30 mmHg in control group, which indicated ATEPH models were successfully established. Between control group and PADN group, the pre-ATEPH values of RVSP, RVDP and mRVP had no statistical significances (*P* > 0.05, Table 1). After injecting thrombus suspension, post-ATEPH RVSP and post-ATEPH mRVP elevations in PADN group were significantly lower than control group (*P* < 0.05, Table 1, Fig. 3), while post-ATEPH RVDP elevations had no difference between two groups (*P* > 0.05, Table 1 and Fig. 3).

**Table 1:** Hemodynamic parameters of ATEPH rabbits ($\overline{x}\pm$ *s*)

	Control (*n* = 6)			PADN (*n* = 5)				
	Preoperative data	Postoperative data	*P*	Preoperative data	Postoperative data	*P*	*P* ^a^	*P* ^b^
RVSP(mmHg)	26.14 ± 7.12	41.01 ± 7.12	0.000	21.91 ± 4.28	29.21 ± 3.25	0.003	0.277	0.008
RVDP(mmHg)	2.21 ± 6.73	7.34 ± 10.98	0.043	-0.25 ± 0.84	4.07 ± 4.03	0.059	0.441	0.544
mRVP(mmHg)	13.19 ± 6.15	23.55 ± 8.29	0.000	9.8 ± 2.62	15.55 ± 2.69	0.005	0.287	0.070

RVSP = right ventricle systolic pressure; RVDP = right ventricle diastolic pressure; mPVP = mean right ventricle pressure; Con = control group; PADN = pulmonary artery denervation group. *P*: comparison in same group before and after establishing ATEPH rabbits. *P *^*a*^: comparison between control and PADN group before establishing ATEPH rabbits. *P *^*b*^: comparison between control and PADN group after establishing ATEPH rabbits

### Ultrasound

Before and after establishing ATEPH rabbit models, all rabbits were examined by transthoracic echocardiography. Between PADN group and control group, RVD, RAD, LAD, LVD, PAD, PAAT and TAPSE had no statistical significance before ATEPH establishment (all *P* > 0.05, Table 2).

**Table 2:** Ultrasonography parameters of ATEPH rabbits ($\overline{x} \pm$ *s*)

	Control (*n* = 6)			PADN (*n* = 5)				
	Preoperative data	Postoperative data	*P*	Preoperative data	Postoperative data	*P*	*P* ^a^	*P* ^b^
RAD (mm)	6.60 ± 1.37	11.29 ± 3.89	0.013	6.87 ± 0.43	9.24 ± 1.97	0.074	0.689	0.315
RVD (mm)	7.21 ± 1.09	10.88 ± 3.71	0.048	6.65 ± 0.43	9.96 ± 2.95	0.055	0.314	0.661
LAD (mm)	7.29 ± 1.01	7.75 ± 1.85	0.469	6.77 ± 1.19	8.08 ± 2.01	0.060	0.459	0.780
LVD (mm)	8.72 ± 1.27	8.75 ± 2.80	0.983	8.82 ± 1.18	8.37 ± 1.66	0.633	0.898	0.795
PAD (mm)	6.23 ± 0.61	7.76 ± 1.13	0.011	5.83 ± 0.41	6.33 ± 0.83	0.149	0.246	0.044
PAAT (mm)	40.83 ± 3.19	27.00 ± 5.59	0.002	37.80 ± 6.06	27.60 ± 1.52	0.008	0.313	0.822
TAPSE (mm)	7.58 ± 2.71	4.60 ± 2.21	0.007	6.42 ± 1.11	4.02 ± 1.72	0.096	0.397	0.641

RAD = right atrium diameter; RVD = right ventricle diameter; LAD = left atrium diameter; LVD = left ventricle diameter; PAD = pulmonary artery diameter; PAAT = pulmonary artery acceleration time; TAPSE = tricuspid annular plane systolic excursion; Con = control group; e PADN = pulmonary artery denervation group. *P*: comparison in the same group before and after establishing ATEPH rabbits. *P *^*a*^: comparison between control and PADN group before establishing ATEPH rabbits. *P *^*b*^: comparison between control and PADN group after establishing ATEPH rabbits

**Fig. 4:**
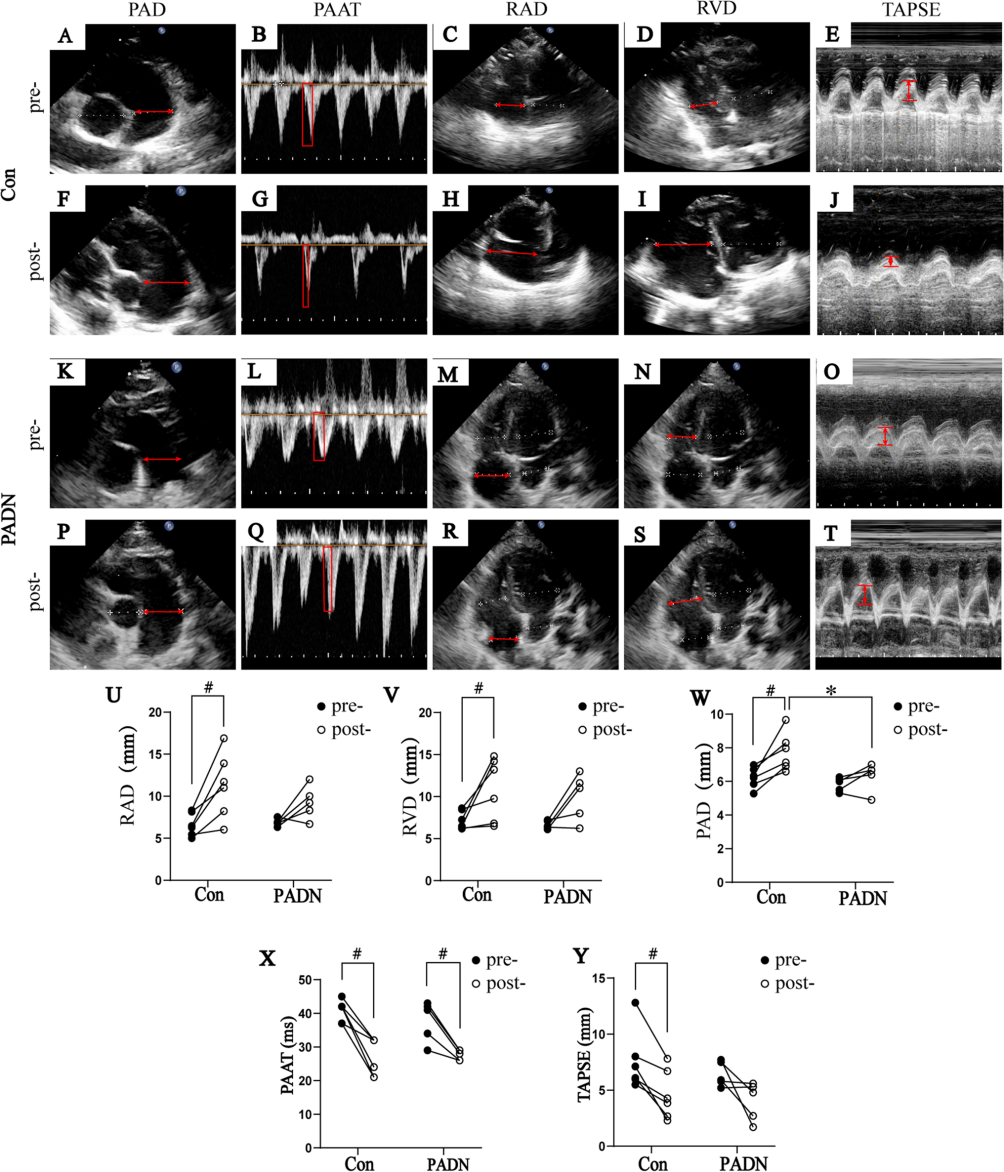
Ultrasonography parameters (**A**) in control group and PADN group before and after establishing ATEPH models. Changes of RAD (**B**), RVD (**C**), PAD (**D**), PAAT (**E**), TAPSE (**F**) between control group and PADN group before and after establishing ATEPH models. PAD = pulmonary artery diameter; PAAT = pulmonary artery acceleration time; RAD = right atrium diameter; RVD = right ventricle diameter; TAPSE = tricuspid annular plane systolic excursion; Con = control group; PADN = pulmonary artery denervation group. Comparisons in same group before and after establishing ATEPH rabbits: ^#^ *P* < 0.05; Comparisons between control group and PADN group: ^*^ *P* < 0.05

After establishing ATEPH models, pulmonary artery forward flow spectrum developed a spike like shape and PAAT in both group became shorter obviously (*P* < 0.05, Table 2). Immediately after the rise in RVSP, TAPSE became significantly lower, while RAD, RVD and PAD dilated significantly in control group, however, LAD and LVD showed no obvious change. In PADN group, TAPSE, RAD, RVD, PAD, LAD and LVD showedno significant change. Compared with PADN group, PAD dilated more significantly in control group (*P* < 0.05), while all other parameters showed no obvious difference (all *P* > 0.05) (Fig. 4).

## Histopathological Examination

### The Histologic Analysis of Lung Tissue with Hematoxylin and Eosin Staining

**Fig. 5:**
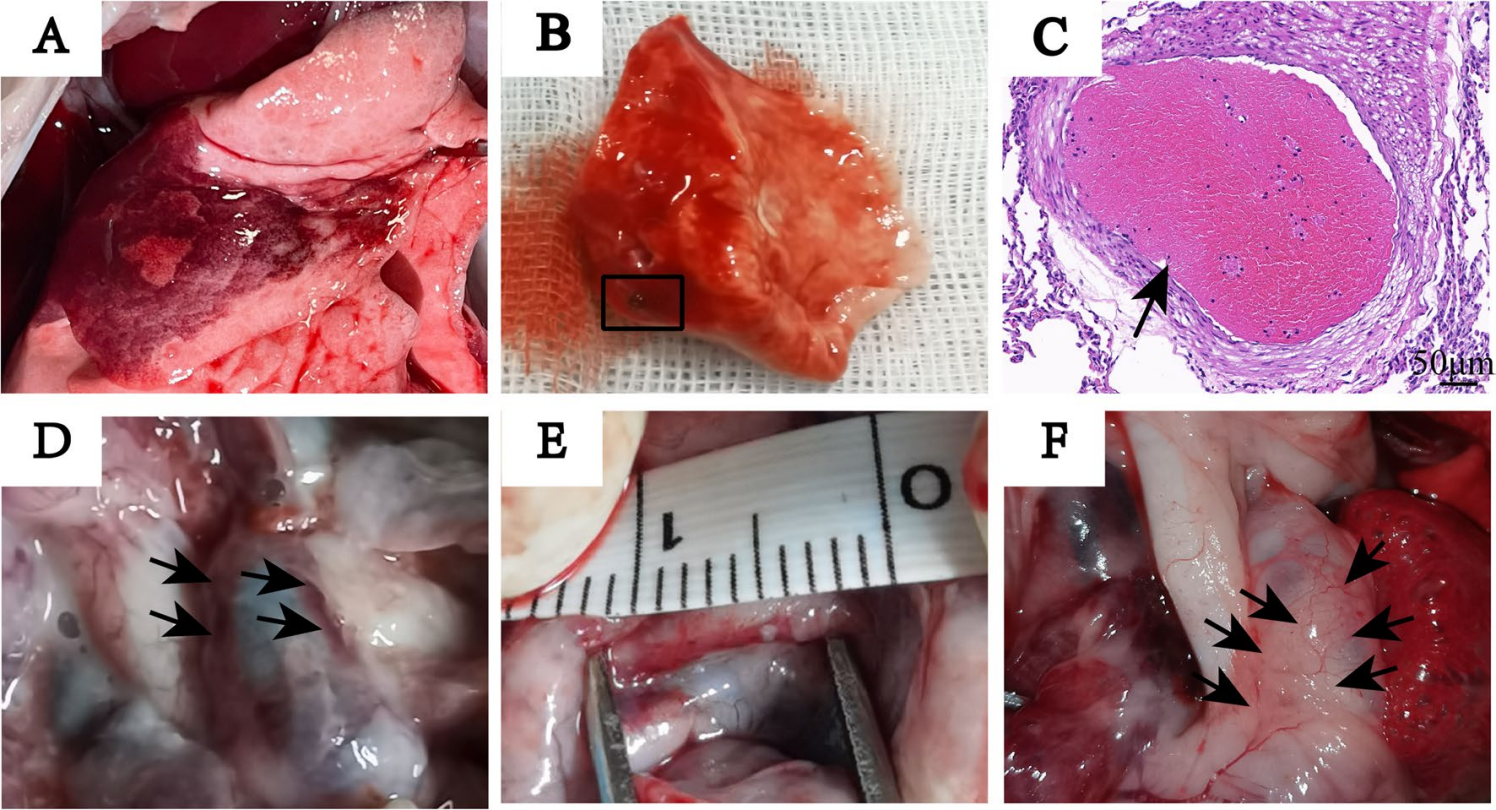
The gross specimen of lung tissue after establishing ATEPH rabbits. (**A**) An obvious segmental infarct in the lung tissue. (**B**) Thrombus existed in the section of infarct of lung tissue (black boxed area). (**C**) HE-stained histological section of lung tissue showed thrombi wedged into an arteriole. (**D**) Arrows indicate linear pale-red ablation foci on the left and right side of MPA adventitia. (**E**) Length of one ablation site was 8 mm. (**F**) Arrows indicate flaky pale-red ablation foci on MPA adventitia

After anesthesia and euthanasia, the lungs were harvested and observed. All ATEPH models were shown obvious segmental infarct in the lung tissue. Wedge-shaped infarct existed from the margin to the bottom of lungs, and normal lung tissue were seen between the infarct. Thrombus could be seen in the section of infarct foci of lung tissue, and the HE-stained histological sections showed thrombi wedged into some small and medium arteries, of which vascular lumens were almost completely blocked (Fig. 5).

### The Distribution of Ablation Sites

There were 5 rabbits in PADN group successfully completed PADN. One rabbit was found pale red ablation foci precisely on the left and right side of MPA adventitia, which was in conformity with target region. Four rabbits were found flaky pale red ablation foci on the adventitia of MPA, however, necrosis ablation foci were also observed in the adjacent lung tissue (Fig. 5). However, ablation zone was only found in right lung of one rabbit.

### Changes of Neural Tissue

**Fig. 6:**
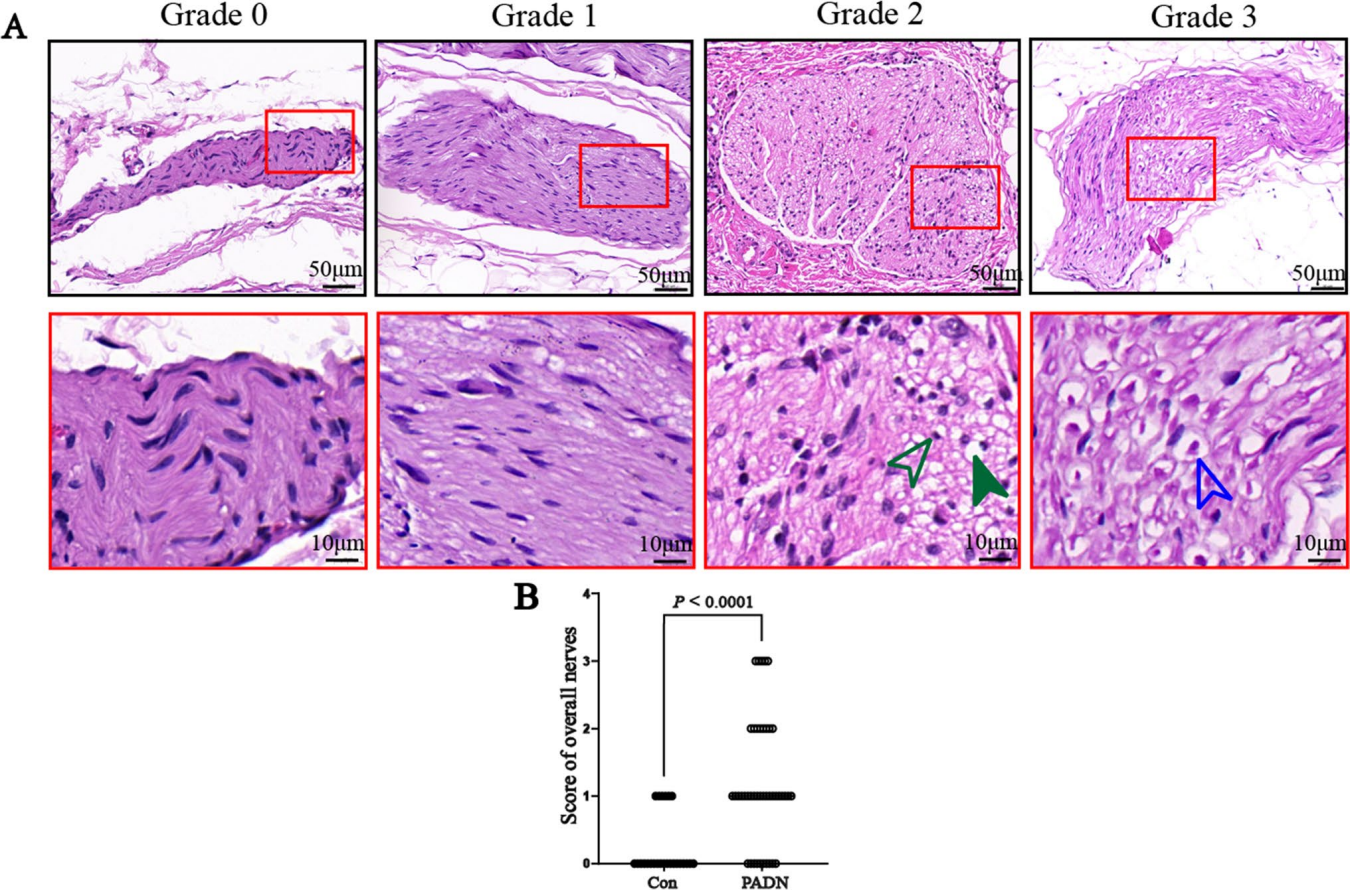
(**A**) Semiquantitative grading scheme for nerve with hematoxylin and eosin (HE) staining. (Upper) Representative images of nerves by increasing grade of injury. (Lower) High-power images of injured nerves (red boxed area in upper panel) in each grade. (Lower) Representative images of vacuolization (green solid arrowhead), pyknotic nuclei (green open arrowhead), digestion chambers (blue open arrowhead). (**B**) Semiquantitative nerve injury scores between control group and PADN group

HE staining showed neuronal nuclei in control group were neatly arranged in oval shape, without vacuolization, pyknotic nuclei or karyolysis and so on. Compared with control group, nerve cells of pulmonary artery adventitia showed obviously damages including vacuolization, pyknotic nuclei and digestion chambers in PADN group. And injured nerve tissue from grade 0 to grade 3 was observed after denervation, and semiquantitative nerve injury scores of PADN group (grade 0–3) was significantly higher than that of control group (grade 0–1) (*P* < 0.0001, Fig. 6).

### Movat-Russell Modified Pentachrome Staining of Pulmonary Artery

**Fig. 7:**
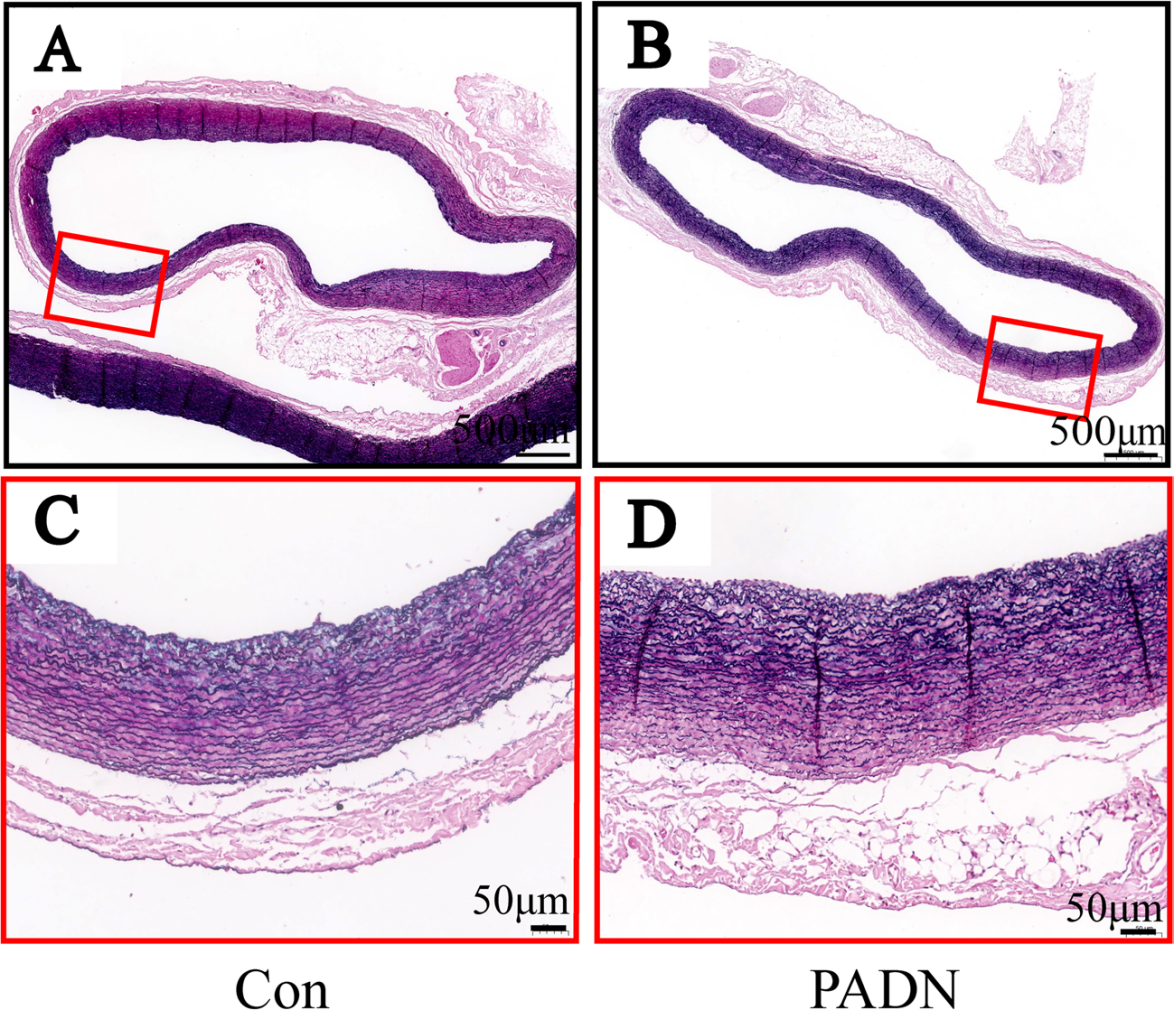
Representative images of pulmonary artery stained by Movat-Russell modified Pentachrome in control group (**A**) and PADN group (**B**). High-power images of pulmonary artery (red boxed area in upper panel) in control group (**C**) and PADN group (**D**). Pulmonary artery was intact without vascular endothelial cell damage or irregular arrangement of PASMCs and there was also no evidence of proteoglycan in place of PASMCs. Con = control group; PADN = pulmonary artery denervation group; PASMCs = pulmonary artery smooth muscle cells

Magnified photomicrographs in both groups showed that the structure of pulmonary artery was intact without vascular endothelial cell damage or irregular arrangement of pulmonary artery smooth muscle cells (PASMCs). There was also no evidence of proteoglycan in place of PASMCs, suggesting no obvious damage to pulmonary artery smooth muscle (Fig. 7).

### Immunohistochemical Staining

**Fig. 8:**
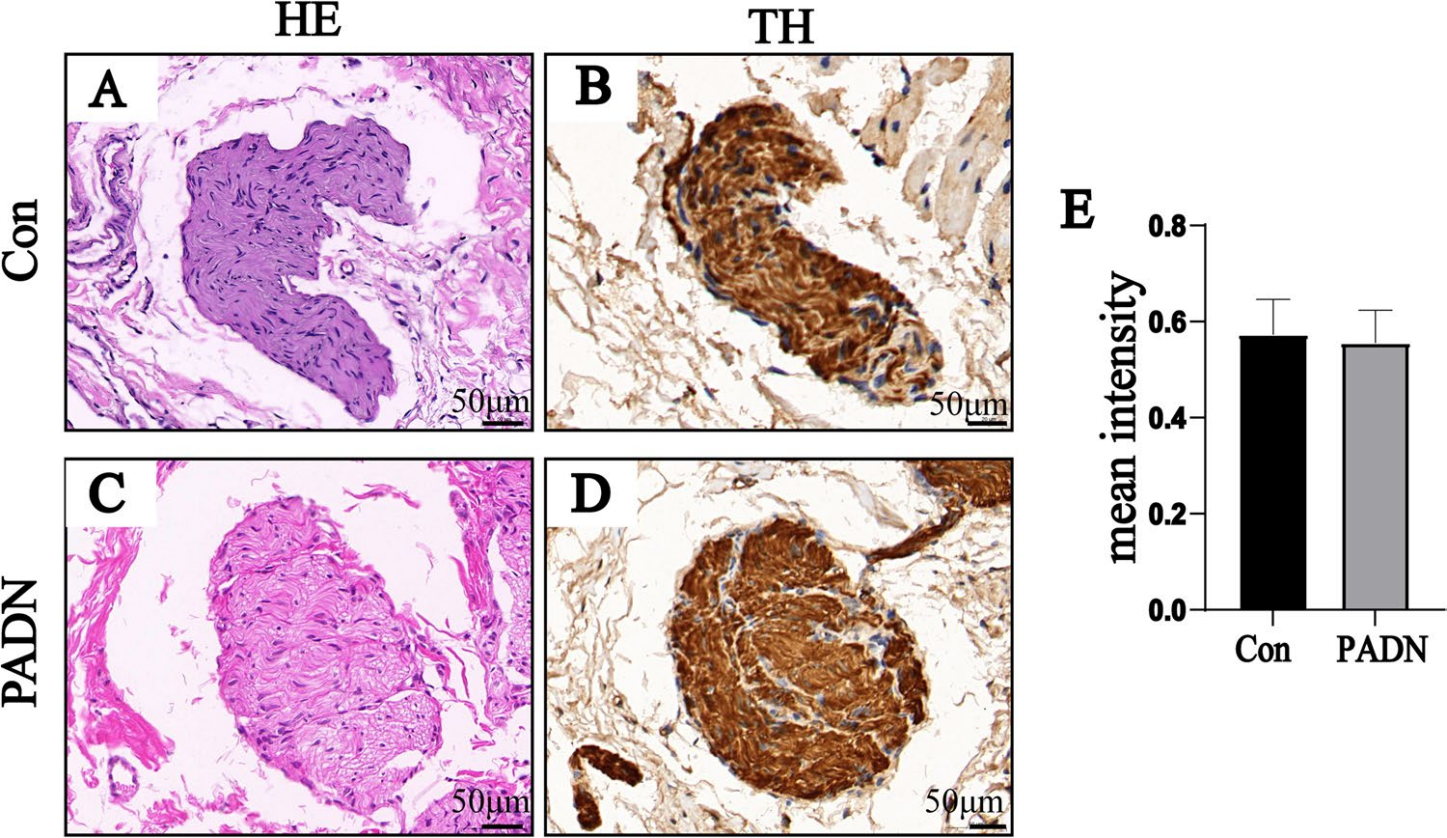
A representative image of nerve with hematoxylin and eosin (HE) staining (**A**) and a corresponding image stained by anti-tyrosine hydroxylase (TH) (**B**) in control group. A representative image of nerve with HE staining (**C**) and a corresponding image stained by anti-TH (**D**) in PADN group. (**E**) Immunohistochemical staining showed mean intensity of TH of all SNs between control group and PADN group. Con = control group; PADN = pulmonary artery denervation group

Immunohistochemical staining showed mean intensity of TH of all SNs had no significant difference between two groups (*P* > 0.05, Fig. 8). Evaluations of immunohistochemical staining against TH demonstrated no thermal effect in PADN group (grade 2–3).

## Discussion

PAH is characterized with increased pulmonary artery pressure and right heart failure [22]. Studies demonstrated that over-activation of sympathetic nerves played a vital role in development of PAH [23, 24], which suggesting sympathetic modulation may be a potential target for treatment of PAH. Preclinical studies have demonstrated that PADN improves pulmonary haemodynamics in acute and chronic models of PAH. Chen et al. found radiofrequency (RF) ablation at the main bifurcation area of the left pulmonary artery could completely abolished the increase haemodynamic in acute PAH animal models during the balloon occlusion of the left pulmonary interlobar artery [25]. Rothman et al. showed that catheter—based PADN induced histological and biochemical alteration and correlated with improved hemodynamic parameters in acute PAH swine model by intravenous infusion of TxA2 [3]. A study also showed RF ablation induces permanent SN injury and subsequent improvements in hemodynamics and PA remodeling in chronic monocrotaline-induced PAH animal models [4]. The preclinical investigations in patients also proved catheter—based PADN could improve functional capacity and haemodynamic parameters in patients with PAH [5, 15] or pulmonary hypertension associated with the left heart disease [9, 16]. However, some limitations were existed in those procedures, including restrictions on suitable candidate screening, local endothelial injury or risk of invasion procedure [10]. Inspired by the operation of HIFU—based renal sympathetic denervation (RDA) effectively disrupting renal sympathetic nerves, this research first-timely explored feasibility and safety of HIFU in PADN, which showed HIFU could be applied in PADN sucessfully.

In our study, hemodynamic and ultrasonography parameters had no significant differences between PADN group and control group before ATEPH, which demonstrated that ablating PA via HIFU had no obvious injuries to cardiac structure and function within a short time. The right ventricular pressure of all rabbits in both group increased immediately after injecting autogeneic thrombus, however, RVSP and mRVP elevations in PADN group were significantly lower than control group, which suggested PADN with HIFU can improve hemodynamics of ATEPH models. Rothman et al. discovered that rising degree of PAP post-PADN was lower than that of pre-PADN in acute pulmonary artery hypertension (APAH) swine models, illustrating PADN could improve hemodynamics in APAH animals [3], which was consistent with our study.

Echocardiography showed that RAD, RVD, PAD dilated and TAPSE decreased after establishing ATEPH models in control group, which were consistent with previous researches [18]. With RV pressure rising, pulmonary artery forward flow spectrum appeared dagger-like immediately, and PAAT became shorter significantly, which were consistent with the changes of PAAT in the chronic pulmonary hypertension rats induced by monocrotaline in previous studies [26]. PAAT lower than 70 ms was a high-risk prediction of PAH in previous studies [27], while in our study, PAAT was 40.83 ± 3.19 ms when RVSP was normal according to right heart catheterization, which may relate to faster heart rate and shorter cardiac cycle of rabbits. Echocardiography evaluated PAP via tricuspid regurgitation velocity [28], however, tricuspid regurgitation was mostly insignificant in ATEPH rabbits in our research, which suggested that it was not feasible to evaluate PAP only by echocardiography and parameter correlation between echocardiography and RHC need further verify in ATEPH models.

HIFU could selectively ablate deep lesions without damage to the adjacent tissues. A previous study showed HIFU-based RDN could effectively and safely reduce blood pressure of experimental canines with energy of 200-250W [10]. In our study, after HIFU procedure, chest skin of rabbits had no obvious injury or only slight redness and swollen. Furthermore, when acoustic energy was 250W, semiquantitative histological assessment of SNs around pulmonary artery in PADN group had higher grade nerve injury scores than controls, and histological evaluation showed no obvious damage in adjacent vascular wall. These results suggested that adventitia SNs were vulnerable to 250 W ultrasonic energy damage, and MPA and bifurcation of PA had a better tolerance to the same ultrasound energy. Nerve fascicle consist of a multilayer lipid membrane structure, which contributed to acoustic energy reflection and deposition within nerve fascicle for many times, proving more possibilities for ultrasound-mediated ablation [10]. In contrast, ultrasonic wave was delivered externally and the rapidly circulating blood stream can cool temperature of pulmonary artery, further avoiding injury to vessel wall [10].

Ablatin foci on the MPA adventitia in PADN group were visible and histological alterations of nerves and PA following PADN with HIFU were consistent with earlier reports of HIFU -base renal sympathetic denervation (RAD) and catheter- based PADN with altered structure. Previous studies had also confirmed that mean-intensity of TH staining of SNs was lower after PADN [17]. However, mean-intensity of TH staining between two groups had no significant difference in our study. The mild, or even no, degradation of TH protein happened in SNs around PA that being harvested at the acute time of PADN procedure, which may result in no significant decline of TH staining.

Over the past years, PADN has achieved some success with catheter-based denervation [29], however, part of PAH patients cannot tolerate invasive PADN procedure [30]. Furthermore, catheter-based devices are invasive and require the use of fluoroscopy and contrast agents, which increase risks and complications for PAH patients [10]. HIFU-based PADN are non-invasive and can monitor the anatomic structure of PA simultaneously, which means patients less susceptible to complication that occurs in intravascular PADN procedure.

After PADN procedures, 5 rabbits were successfully conducted PADN, of which the ablation zone was also observed in right auricle or right lung in 4 rabbits and ablation zone was detected only in right lung in 1 rabbit. PA is adjacent to lung and heart, respiratory and cardiac beat can result in movement of target, nevertheless, transducer of HIFU is fixed and absents auto-focus system, which affect the accuracy of ablation. Tzafriri et al. found arterial microenvironment could influence ablation effect when performing catheter-based RDN [31]. During radiofrequency ablation, peak energy intensity of RDN could be shifted by tissues around arteries, especially tissues with high water content, such as lymph nodes, which could significantly interfere direction of energy propagation. We speculate these tissues might also alter direction of ultrasound energy when conducting PADN with HIFU. During performing HIFU procedure, in order to concentrate ultrasonic energy on PA, ultrasonic transducer should be kept at a fixed distance from ablating target and degassed water was used to help maintain the distance between transducer and skin of rabbit. As a result, resolution ratio in ultrasound image would decrease, which brought difficulty in accurately focusing target. Thus, for the later application of HIFU—base PADN in a beating heart, the creation of a bilateral artificial pleural effusion to push the lung away from the heart, and breath control may help to reduce the damages to the lung. Morever, it will be necessary to improved the resolution ration and accurate adjustment of diagnostic transducer, explore auto-focus system precisely aim the targeted PA, even develop new software incorporated electrocardiographic gating to synchronize HIFU exposures with each cardiac cycle.

### Limitation

Acute PAH animal models were established by acute pulmonary thromboembolism without gradually inducing pulmonary vascular remodeling, which might not exactly consistent with progressive PAH in clinical. In this study, we only examined hemodynamics and histological analysis within 12 h after PADN, however, nerve regeneration may occur after PADN procedure, so that long-term curative effect needs further study.

## Conclusion

In this study, we firstly demonstrated that HIFU could acutely destroy SNs around PA effectively and safely, which may be a new choice to conduct PADN. However, the long-term hemodynamic effects of PADN with HIFU needs further study and the accuracy of HIFU in PADN needs to be improved.

## Data Availability

The data underlying this article will be shared on reasonable request to the corresponding author.
